# Development of an in vitro regeneration system from immature inflorescences and CRISPR/Cas9-mediated gene editing in sudangrass

**DOI:** 10.1186/s43141-023-00517-6

**Published:** 2023-05-15

**Authors:** Shireen K. Assem, Mahmoud A. Basry, Taha A. Taha, M. H. Abd El-Aziz, Taher Alwa, Walid M. Fouad

**Affiliations:** 1 Department of Plant Molecular Biology, Agricultural Genetic Engineering Research Institute (AGERI), Agricultural Research Center (ARC), Giza, Egypt; 2 Genetics Department, Faculty of Agriculture, Mansoura University, Mansoura, Egypt; 3 Department of Biology, School of Science and Engineering, American University in Cairo, New Cairo, 11835 Cairo Egypt

**Keywords:** Sorghum, Sudangrass, Genome editing, CRISPR/Cas9, Caffeic acid *O*-methyltransferase (COMT) gene, Immature inflorescence, Biolistic transformation

## Abstract

**Background:**

Sudangrass (*Sorghum sudanense*) is a major biomass producer for livestock feed and biofuel in many countries. It has a wide range of adaptations for growing on marginal lands under biotic and abiotic stresses. The immature inflorescence is an explant with high embryogenic competence and is frequently used to regenerate different sorghum cultivars. Caffeic acid *O*-methyl transferase (COMT) is a key enzyme in the lignin biosynthesis pathway, which limits ruminant digestion of forage cell walls and is a crucial barrier in the conversion of plant biomass to bioethanol. Genome editing by CRISPR/Cas9-mediated mutagenesis without a transgenic footprint will accelerate the improvement and facilitate regulatory approval and commercialization of biotech crops.

**Methods and results:**

We report the overcome of the recalcitrance in sudangrass transformation and regeneration in order to use genome editing technique. Hence, an efficient regeneration system has been established to induce somatic embryogenesis from the immature inflorescence of two sudangrass cultivars on four MS-based media supplemented with different components. Our results indicate an interaction between genotype and medium composition. The combination of Giza-1 cultivar and M4 medium produces the maximum frequency of embryogenic calli of 80% and subsequent regeneration efficiency of 22.6%. Precise mutagenesis of the COMT gene is executed using the CRISPR/Cas9 system with the potential to reduce lignin content and enhance forage and biomass quality in sudangrass.

**Conclusion:**

A reliable regeneration and transformation system has been established for sudangrass using immature inflorescence, and the CRISPR/Cas9 system has demonstrated a promising technology for genome editing. The outcomes of this research will pave the road for further improvement of various sorghum genotypes to meet the global demand for food, feed, and biofuels, achieving sustainable development goals (SDGs).

## Background

Global food security is adversely affected by population growth, which also contributes to the current energy crisis. Sorghum is a versatile crop that can be grown in arid and semi-arid regions where other economic crops cannot thrive. It is used as a grain supply and a source of forage, fiber, fuel, and secondary products. Sorghum is the fifth most significant cereal crop across the globe [1]. It is considered a strategic crop, especially for food and livestock feed in Africa, Asia, South America, and the USA [2]. In the present time, sorghum genotypes have a diverse range of varieties with obvious potential in various applications [3]. The genus Sorghum includes 24 species, of which few are cultivated [4]. Sudangrass is one of the important species of sorghum, which is primarily used as feed and fodder for animals and used to produce biofuel from lignocellulosic feedstock [5]. Sudangrass is also drought-tolerant and climate-change resilient, as it is a C4 plant that is highly efficient in water use and biomass production [6, 7].

The first successful transgenic sorghum plants were obtained through particle bombardment by Casas et al. [8]. Sorghum species are recalcitrant for in vitro culture regeneration and genetic transformation due to their vast diversity, which is mostly due to the release of toxic phenolics in the culture medium, a lack of regeneration in long-term in vitro cultures, and a high degree of genotype dependence [3, 9, 10]. As a result, the transformation of cells followed by plant regeneration represents a bottleneck for molecular improvement of forage sorghum, while the system is well established for a few grain sorghum genotypes [11, 12]. So, establishing sudangrass regeneration systems based on somatic cells constitutes a crucial requirement in the development of the process of molecular improvement of multipurpose sudangrass varieties [13, 14].

Successful callus/somatic embryogenesis and subsequent plant regeneration have been reported from various sorghum explants, such as immature inflorescences [12, 15, 16], shoot tips or meristems [9, 17, 18], leaf segments [10, 19], and floral organs [20]. At the same time, immature zygotic embryos were successfully utilized in many grain varieties [21, 22]. However, immature inflorescences and immature zygotic embryos have the highest embryogenic competency and are frequently used to regenerate several sorghum varieties [15]. Furthermore, it has been reported that growing donor plants to produce immature inflorescence is faster than growing immature embryos, which have a higher proportion of meristematic tissues and a better potential for regeneration [16, 23]. The present status of in vitro tissue culture techniques of sudangrass is insufficient due to the low efficiencies of callus induction and plant regeneration compared with those other cereals [24, 25]. Furthermore, the release of phenolics in culture inhibits callus growth and somatic embryogenesis in immature inflorescences-derived callus cultures of elite sudangrass varieties for the production of a high number of regenerated plants [12, 22].

Application of genome editing technologies in sudangrass is lagging behind other cereal crops and is limited to a few *S. bicolor* grain varieties. The first report of CRISPR/Cas9-mediated gene editing in sorghum using *Agrobacterium*-mediated transformation was reported in 2013 [26]. Since then, a handful of reports on improving the genome editing of sorghum have been published [27–29] using phenotypic traits and morphogenic genes. To our knowledge, there are no reports on genome editing of sudangrass using CRISPR/Cas9 system [1, 30].

Lignin, on the other hand, is a heterogeneous phenolic polymer primarily consisting of p-hydroxyphenyl (H), guaiacyl (G), and syringyl (S) units that are polymerized by the incorporation of various monolignol precursors: p-coumaryl, coniferyl, and sinapyl alcohol, respectively [31]. Lignin is one of the cell walls’ main components and a significant factor limiting the efficient bioconversion of lignocellulosic biomass through biological digestion and chemical degradation [32]. Lignin impairs the hydrolysis of the polysaccharides into their monomeric sugars in ruminant livestock or cellulosic bioenergy [6, 33]. The complexity of the lignin biosynthetic pathways is attributed to several multi-functional enzymes, and these enzymes also correspond to diverse gene families [34]. Subsequently, several studies reported the potential of engineering key enzymatic steps in lignin pathways, such as the caffeic acid *O*-methyltransferase (COMT) gene [EC 2.1.1.68] to reduce lignin concentration and/or alter lignin composition [35–37]. Reports of genetic modification of the lignin biosynthetic pathway by downregulation and perturbation of the COMT gene are straightforward strategies for producing transgenic plants with reduced lignin content and normal growth rates. Altered lignin composition improved forage quality due to higher digestibility, increased saccharification efficiency, and increased ethanol production yield [38, 39].

In this report, we demonstrate the successful development of an in vitro regeneration system and the generation of transgenic plants via microprojectile bombardment using immature inflorescences for two sudangrass cultivars. Additionally, successful CRISPR/Cas9 expression and targeted genome editing for the COMT gene are reported.

## Methods

### Biological material

All local and international guidelines were adhered to in this research. Seeds from two forage sudangrass varieties, Giza-1 and Giza-2, were obtained from the Forage Department, Field Crops Research Institute (FCRI), Agricultural Research Center (ARC), Giza, Egypt. Seeds were grown in the Agricultural Genetic Engineering Research Institute (AGERI) experimental field (latitude 26° 31′ N and longitude 31° 11′ E). Seeds of sudangrass were sown in the field with a 3-week interval between each sowing date within the summer season, to provide a continuous source of immature inflorescences. Normal sudangrass cultural practices were applied as recommended in the district by the Field Crops Research Institute (FCRI), such as hoeing, fertilization, and irrigation, prior to the appearance of the flag leaf, and shoots containing unmerged inflorescences (length: 1–5 cm) were taken from the field-grown sudangrass plants. The outermost leaf blades were removed, and the shoots were rinsed in 70% ethanol followed by washing with sterile water by autoclaving. The immature inflorescences were then dissected and split into 5-mm-long pieces as described by Brandão et al. [15].

### Callus induction and regeneration

Four tissue culture media, i.e., M1, M2, M3, and M4, were tested for callus induction from the immature inflorescence of the two cultivars of sudangrass. All mediums contained full-strength MS basal medium [40], with different concentrations of hormones and growth regulators. The pH of the medium was adjusted to 5.8 before adding 3 g/L phytagels and sterilized by autoclaving for 20 min at 121°C and 15 psi. All cultures were incubated at 25ºC ± 2ºC in the dark for 4 weeks with one subculture, after which embryogenic calli were cultured on shoot induction medium (S) for 2 weeks and incubated at 25ºC ± 2ºC with a 16-h photoperiod. Regenerated shoots at least 2 cm in length were transferred to the rooting medium (R) for shoot elongation and rooting. Cultures were maintained at 25ºC ± 2ºC with a 16-h photoperiod. Cool white fluorescent lamps provided light at a photon flux density of 30 µMolm-2s-1. Components of callus induction and regeneration media are presented in Table 1.

**Table 1:** Callus induction and regeneration media used for the optimization of the regeneration system of sudangrass

Component	Callus induction media^*^				Regeneration media^**^	
	M1	M2	M3	M4	Shooting (S)	Rooting (R)
MS basal (g/L)	4.4	4.4	4.4	4.4	4.4	4.4
MES (mg/L)	500	0	0	500	0	0
CuSO_4_ (mg/L)	0	800	0	0	0	0
Glycine (mg/L)	0	0	0	7	0	0
L-proline (mg/L)	700	700	2400	0	700	700
L-asparagine (mg/L)	0	0	0	100	0	0
Myo-inositol (mg/L)	100	150	0	100	100	100
Nicotinic acid (mg/L)	0.5	0	0	1	0.5	0.5
Pyridoxine-HCl (mg/L)	0.5	0	0	1	0.5	0.5
Thiamine-HCl (mg/L)	10	0	0	1	2.5	2.5
Ascorbic acid (mg/L)	0	1	0	0	10	10
2,4-D (mg/L)	1.5	1	4	2.5	0	0
IAA (mg/L)	0	0	0	0	0	1
kinetin (mg/L)	0	0	0.5	0.5	1	1
Zeatin (mg/L)	0	0	0	0	0.5	0
TDZ (mg/L)	0	0	0	0	0.1	0
BAP (mg/L)	0	0.5	0	0	0	0
ABA (mg/L)	0	0	0	0	0.25	0
Charcoal (mg/L)	0	0	0	0	400	400
PVP (mg/L)	0	0	0	0	300	300
Peptone (g/L)	0	0.82	0	0	0	0
Maltose (g/L)	0	30	0	0	0	0
Sucrose (g/L)	20	0	45	20	30	30

^*^Callus induction media: M1 [22], M3 [30], and M4 modified [8]

^**^Regeneration media: (S and R) [22]

### CRISPR/Cas9 vector construction

The CRISPR/Cas9 vector targeting the COMT gene was designed and constructed by Dr. Fredy Altpeter, IFAS-UF, USA. Briefly, in order to design gRNA targeting the COMT gene, the CRISPOR (http://crispor.tefor.net) tool was used. The COMT sequence belonging to sorghum was obtained from the Plant Ensembl Database (SORBI_3007G047300). A highly conserved region in COMT was targeted, which is highly conserved in sugarcane, maize, bahiagrass, and sorghum. The best gRNAs were selected with CRISPOR and evaluated following custom gene synthesis with an in vitro cleavage assay. Two guide RNA (gRNA) sequences were designed based on the corresponding target sites in the COMT locus, i.e., gRNA-392 targeted COMT_Exon1 (COMT1) and gRNA-79 targeted COMT_Exon2 (COMT2).

**Fig. 1:**
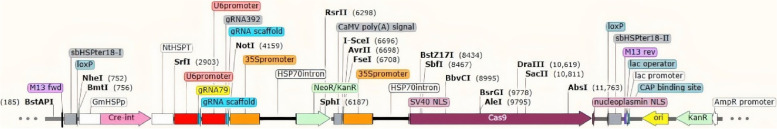
Sorghum CRISPR/Cas9-SbCOMT vector schematic diagram for stable transformation. The plant expression cassette carries the nptII selection marker, which confers resistance to kanamycin or G418 (Geneticin®) in bacteria, and is utilized to select transformed calli and plantlets throughout the selection and regeneration processes

The dual gRNA cassettes were cloned into the plant expression vector to create the final vector (CRISPR/Cas9-SbCOMT) (Fig. 1) with two monocistronic gRNA expression cassettes, a selectable marker expression cassette, a Cas9 nuclease expression cassette, and a heat-inducible Cre expression cassette for excision from the genome following gene transfer via recombination between the flanking loxP sites. The constructed vector was kindly provided by Dr. Altpeter for the transformation of sudangrass with the aim of optimizing gene-editing conditions and knock-out the COMT gene in forage sudangrass for improving forage quality.

### DNA delivery, selection, and regeneration of putatively transgenic plantlets

Embryogenic calli were chosen as targets for particle bombardment. The embryogenic calli were placed on callus-induction medium (M4) supplemented with 45 g/L mannitol and 45 g/L sorbitol for osmotic pretreatment. Calli provided osmotic treatment for 4 h before the bombardment and for 16 h thereafter.

Plasmid DNA was precipitated onto gold particles (Bio-Rad 0.6µm in diameter) using the Bio-Rad Biolistic PDS-1000/He Particle Delivery System’s original protocol. In the transformation experiments, the bombardment parameters were 1100 psi pressure, a distance of 9 cm, and one shot per plate. Following bombardment and osmotic treatment, the plates were placed in callus-induction medium (M4) for a 1-week recovery period in the dark to allow the transformed cells to heal.

Transferring the transformed calli to callus-induction media (M4) containing 10 mg/L Geneticin as a selective agent for 2 weeks was used to select the transformed events for the NPTII. In subsequent subcultures, the concentration of Geneticin was raised to 20 mg/L.

For regeneration, the Geneticin-resistant calli, which grew uniformly on the selection medium, were transferred to the shoot induction medium (S) for 2–4 weeks, followed by transferring the regenerated shoots to the medium (R) for root formation and further development. Putatively transgenic T0 plantlets were acclimatized in the greenhouse by transferring them to pots containing a peatmoss:soil:sand mixture with a ratio of (1:1:1) adjusted at 28°C with a 16-h photoperiod and 50% humidity. For the first 2 weeks, pots were watered with half-strength MS solution, followed by regular water irrigation.

### Molecular analysis of putative transgenic T0 plants

- a. Plant DNA extraction and polymerase chain reaction (PCR) analysis
  The selection of primary transgenic T0 plants. Genomic DNA was isolated from leaf tissues of regenerated plants using the DNeasy® Plant Mini Kit-QIAGEN (Cat. No. 69104) method. The presence of the 35S promoter, NPTII, and Cas9 genes was initially detected by PCR analysis and compared with wild type control (WTC), positive amplification control (PAC), and negative template control (NTC). The 123bp coding region of the 35S promoter gene was amplified using the primers (5′-CCA CGT CTT CAA AGC AAG TGG-3′ and 5′-TCC TCT CCA AAT GAA ATG AAC TTC C-3′). The 224bp coding region of the NPTII gene was amplified using the primers (5′-TTG GGT GGA GAG GCT ATT CG-3′ and 5′-CTT CCC GCT TCA GTG ACA AC-3′). The 260bp coding region of Cas9 nuclease gene was amplified using primers (5′-AGG TGG AGA AGG GAA AGT CG-3′ and 5′-AGT TCA CGT ACT TGG ACG GC-3′). Each of the 25 uL amplification reactions contains 50 ng of template DNA, 10 µM each primer, and 13 µL EmeraldAmp GT PCR Master-Mix (Cat. No. RR310A), which have a 3'-terminal adenosine (A), and therefore, PCR products can be used directly for cloning. The Master-Mix was carried out using a thermal cycler (BioRad-T100, California, USA) under the following conditions: 95°C for 5 min, 30 cycles at 95°C for 30 s, 60.5°C for 30 s, 72°C for 60 s, and a final extension at 72°C for 5 min. The amplified products were separated by electrophoresis on a 1.2% agarose gel and visualized with 0.005% ViSafe Red Gel Stain (Cat. No. SD0103).
- b. Sequencing COMT gene in transgenic sudangrass carrying the CRISPR/Cas9-SbCOMT vector to evaluate the targeted edits

Primers were designed to amplify the targeted COMT gene in transgenic sudangrass carrying the COMT1 and COMT2 editing constructs CRISPR/Cas9-SbCOMT to identify the nature of the mutation introduced by gene editing in each event. PCR products were amplified from the targeted genomic region in sudangrass -Giza-1 T0 and control (non-transformed) cells using primers for COMT1 (5′-GCT CTC CGG CCC CAT ATA AC-3′ and 5′-AGC TAG TAC TAA TGA GCG AGC-3′), gRNA-392 (5′-CGC GCT CAT GAA CCA GGA CA-3′), and COMT2 (5′ PCR has been carried out using a thermal cycler under the following conditions: 95°C for 5 min, 26 cycles at 95°C for 45 s, 62°C for 30 s, 72°C for 60 s, and a final extension at 72°C for 5 min). The purified PCR products were submitted for Sanger sequencing, and the sequences were aligned with the COMT sequences of the control (non-transformed) using the multiple sequence alignment (MSA) method by the “Clustal Omega program.”

### Statistical analysis

Statistical analysis was performed according to IBM (2009) using SPSS software. The experiments were designed as factorial experiments in a completely randomized design with three repetitions. The means were compared by a *t* test at a level of 5% probability.

## Results

### Immature inflorescence in vitro culture and plant regeneration

**Fig. 2:**
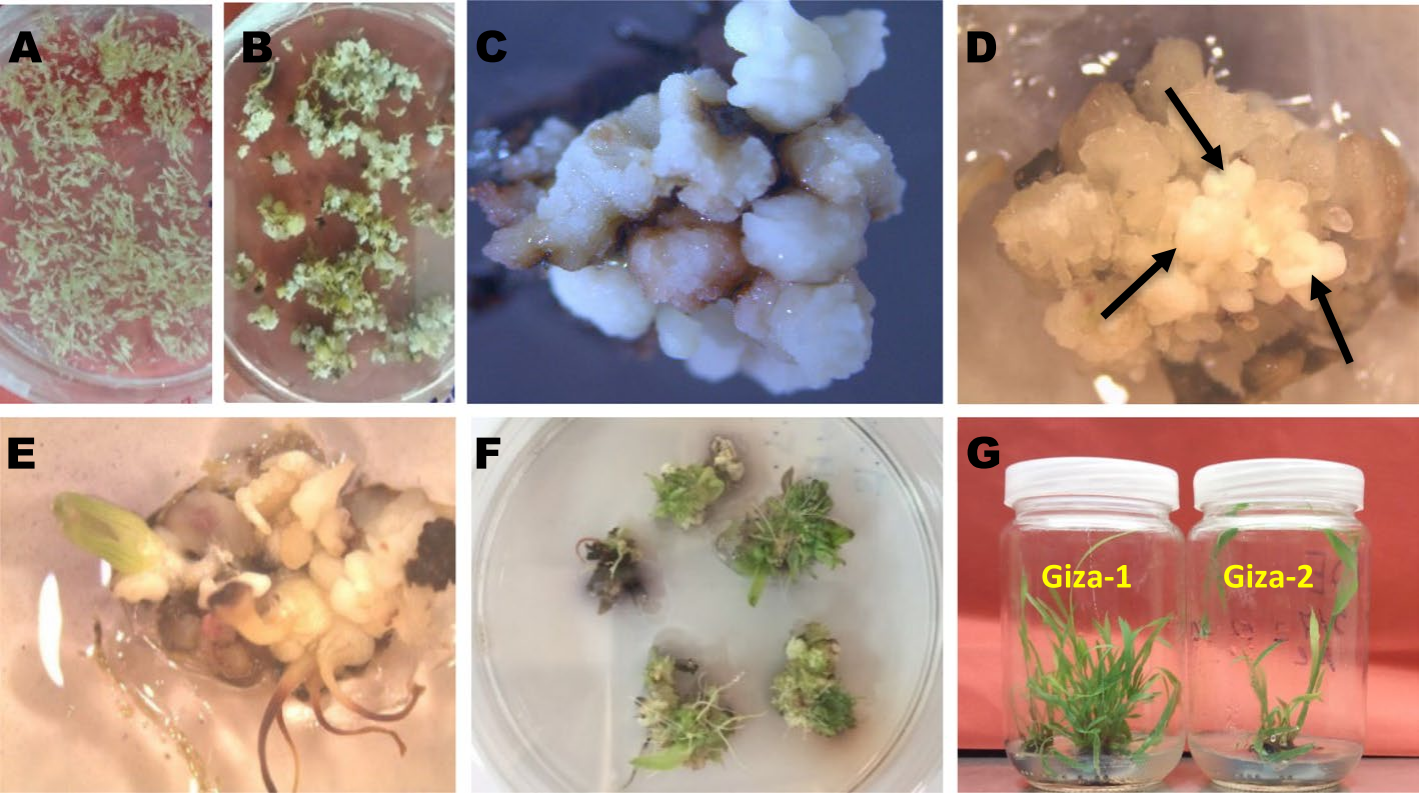
Stages of callus induction and regeneration in the two forage sudangrass Giza-1 and Giza-2. **A** Immature inflorescence dissected and cultured on callus-induction medium. **B** Embryogenic callus formed after 4 weeks. **C** Compact callus. **D** Embryogenic friable callus. **E**, **F** Regenerated shoots. **G** Regenerated plantlets

During initial experiments, we observed that young and small immature inflorescence (2–4 cm) coupled with frequent subcultures every 7 to 10 days greatly enhanced callus induction and reduced browning due to phenolic compounds (Fig. 2 A and B). The two sudangrass cultivars (Giza-1 and Giza-2) were evaluated for the quality of callus formed after 4 weeks in immature inflorescence in vitro culture on the four callus-induction media (M1, M2, M3, and M4). Observations of callus initiation showed the formation of two types of callus, i.e., embryogenic friable and compact calli (Fig. 2C and D). Giza-1 showed a higher rate of proliferation and embryogenic capacity while substantial tissue browning in Giza-2 prevented embryogenesis. Moreover, out of the four investigated callus-induction media, M4 was observed to be superior to the other media for producing embryogenic callus in both cultivars. All embryogenic calli produced on callus induction media were counted and transferred onto the regeneration medium (Fig. 2E, F, and G), and the regeneration frequency was calculated after 4 weeks.

For the callus induction experiments, 100 immature inflorescences were used as explants in 3 replicates for each genotype. The mean percentage of embryogenic calli was calculated as the percentage of calli showing embryogenesis/the total number of explants that showed callus ×100. Furthermore, the regeneration frequency from both cultivars was calculated as the percentage of shoot formation/the percentage of embryogenic calli ×100.

Results presented in Table 2 reveal that media M4 and M3 had a higher potential for producing embryogenic calli from the two cultivars than media M1 and M2. The key difference between these media was that M3 and M4 contain kinetin (0.5 mg/L), while M1 and M2 do not. Furthermore, the M1 and M2 media have low concentrations of 2,4-D (1.5 mg/L and 1 mg/L, respectively), whereas the M3 and M4 media have elevated concentrations of 2,4-D (2.5 mg/L and 4 mg/L, respectively).

**Table 2:** Effect of media composition on the percentage of callus induction from sudangrass (Giza-1 and Giza-2)

**Media**	**Giza-1**		**Giza-2**	
	**Embryogenic calli frequency %**	**Regeneration frequency %**	**Embryogenic calli frequency %**	**Regeneration frequency %**
**M1**	15.00_aD_	0.004_aD_	10.00_bD_	0.00_bD_
**M2**	25.00_bC_	3.80_aB_	30.00_aC_	2.00_bB_
**M3**	45.00_aB_	0.86_aC_	40.00_bB_	0.204_bC_
**M4**	80.00_aA_	22.60_aA_	65.00_bA_	14.70_bA_

Means with different letters within the same row (lowercase) and column (uppercase) differ significantly at *α*=0.05

The highest significant mean percentage of embryogenic callus induction was observed from Giza-1 and Giza-2 immature inflorescences cultured on M4 medium (80_aA_% and 65_bA_%, respectively) compared to the other media. Moreover, the highest mean regeneration frequency was recorded by calli maintained on M4 (22.6% for Giza-1 and 14.7% for Giza-2, respectively).

### Transformation, selection, regeneration, and molecular analysis of putative transgenic plants

**Fig. 3:**
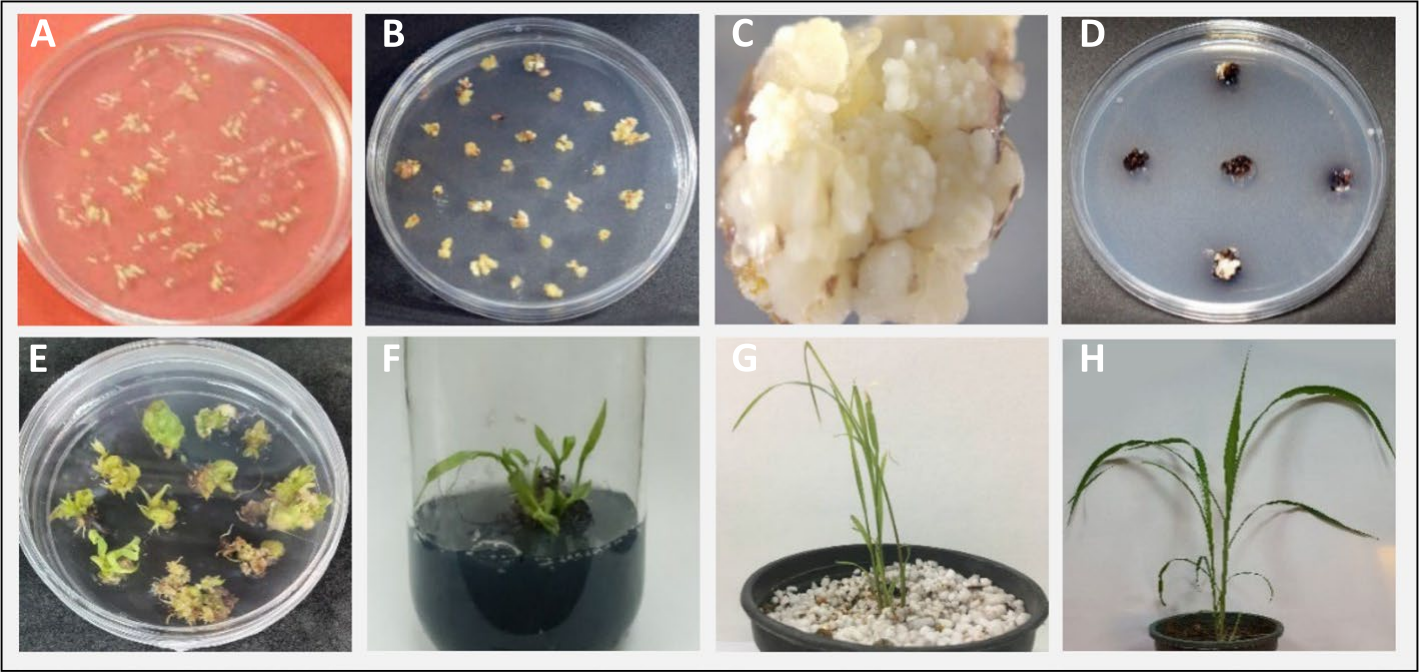
Callus induction, transformation, regeneration, and acclimatization stages. **A** Immature inflorescence after dissection. **B** Callus induction. **C** Embryogenic callus for bombardment. **D** Transformed cells on selective medium. **E**, **F** Shoot induction on regeneration medium. **G**, **H** Acclimatization and plant development in the greenhouse

A total of 2674 sudangrass Giza-1 embryogenic calli were bombarded with the CRISPR/Cas9-SbCOMT vector and selected on Geneticin of which 1087 calli survived on selection media and were transferred to regeneration media (Fig. 3).

### PCR analysis

**Fig. 4:**
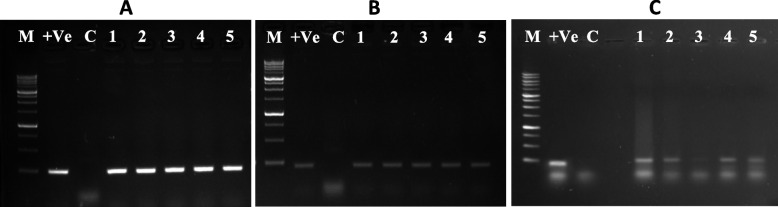
PCR reactions carried out for the Cas9 ∼260bp (A) and NPTII ∼224bp (B) genes and 35S promoter ∼189bp (C) in transgenic T0 plants. (M) 1kb molecular weight size marker; (C) control (nontransformed plant); (+Ve) positive amplification control of the vector; Lanes (1:5) are DNA samples from individual events sudangrass resulting from transformation by biolistic particle delivery systems

PCR reactions have been carried out to detect Cas9 and NPTII gene and the 35S promoter as elements of the transformation vector in the transgenic plants. The presence of the bands corresponding to Cas9 (260 bp), NPTII (224 bp), and 35S-P (189 bp) confirmed the successful transformation of the regenerated transgenic plants (Fig. 4). A number of 98 shoots have been recovered and regenerated. PCR analysis detected 30 positive Cas9, NPTII, and 35S promoter events. The transformation efficiency was 30.6% (no. of Cas9 transgenic events/no. of Geneticin-resistant events*100).

### DNA sequencing analysis

**Fig. 5:**
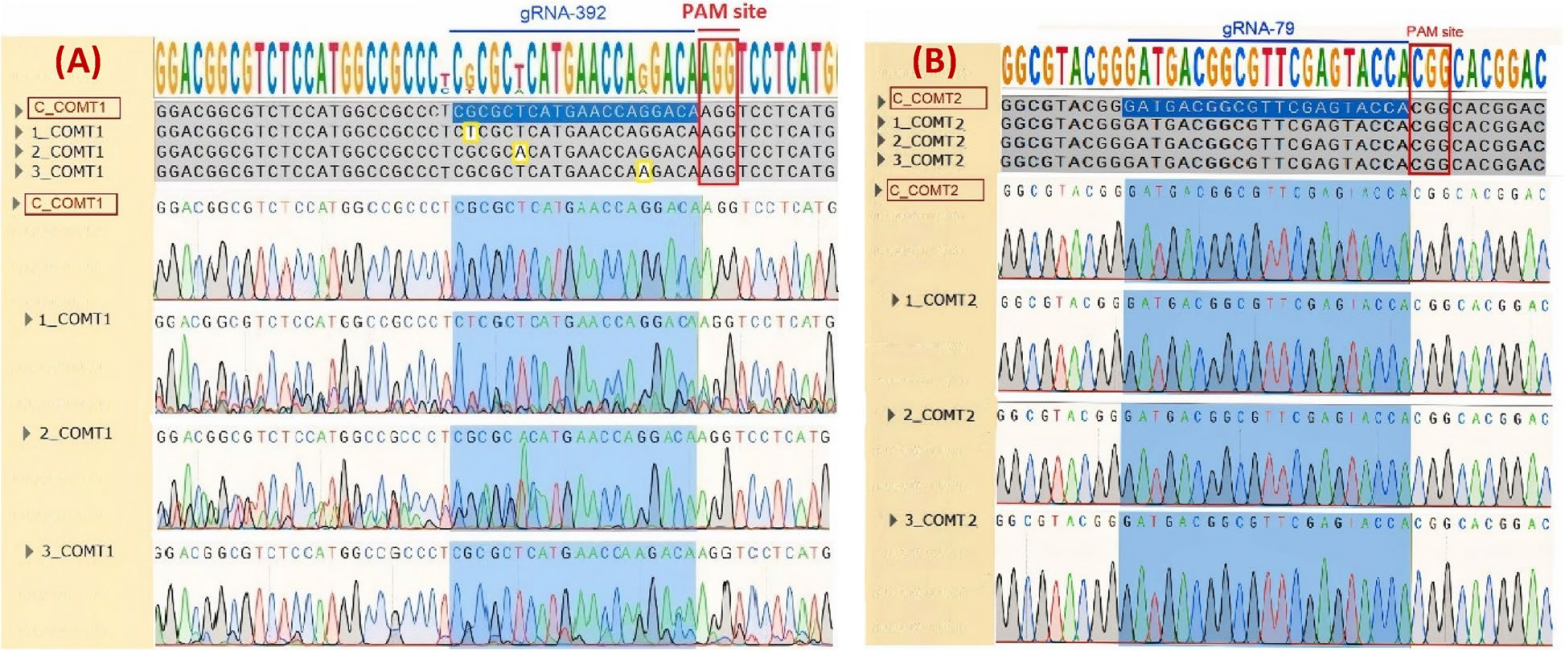
Sanger sequencing electropherogram of purified PCR products from the target sequence of the CRISPR/Cas9 system. A Sequencing was carried out using COMT_Exon1 primers to target gRNA-392; C_COMT1 is a control plant negative for the CRISPR/Cas9 system, while 1_COMT1, 2_COMT1, and 3_COMT1 are transgenic plants positive for the CRISPR/Cas9 system. B Sequencing was carried out using COMT_Exon2 primers to target gRNA-79; C_COMT2 is a control plant negative for the CRISPR/Cas9 system, while 1_COMT2, 2_COMT2, and 3_COMT2 are transgenic plants positive for the CRISPR/Cas9 system. The red border denotes the PAM site, and the yellow border denotes the gRNA target site substitution. Sequencing chromatograms indicate the mutation in the target sequences, resulting in the variety of peaks in the chromatograms. The position of the gRNA is highlighted in blue

The alignment of 30 purified PCR samples of COMT gene sequences from sudangrass targeted with the CRISPR/Cas9-SbCOMT vector compared with the control showed nucleotide mutations in the form of substitutions in COMT1 in three events. Otherwise, there was no nucleotide mutation in COMT2 (Fig. 5). The editing efficiency was estimated at 10% (no. of edited plants/no. of Cas9-positive plants * 100).

## Discussion

An efficient method of plant regeneration is necessary for applying genetic manipulation technologies in any crop improvement program. Reports of plant regeneration from sudangrass tissue cultures are very limited due to the recalcitrance and phenol secretion in the cultures [5, 13]. This study is one of the very few reports describing the development of a reliable regeneration system for sudangrass (*S. sudanense*) *via* somatic embryogenesis from immature inflorescence explants. Regarding the efficiency of embryogenic callus formation, Giza-1 was significantly more efficient than Giza-2 on all the tested media, showing that callus induction in sudangrass is genotype dependent. Among the four tested callus induction media, M4 was the most efficient medium for embryogenic callus induction, and callus maintained on M4 had a significantly higher regeneration frequency in comparison to callus maintained on the other media, showing that the composition of callus induction medium had a great influence on the type and number of callus produced, and consequently on the callus regeneration capacity. The effect of genotype × medium interaction has been shown by the highly significant number of friable embryogenic callus produced by Giza-1 in comparison to Giza-2 on M4 medium. Our findings were consistent with previous sorghum tissue culture research, which revealed that callus induction and regeneration are genotype-dependent, with a genotype-callus induction medium composition interaction [16, 25, 41].

2,4-D has been the most commonly used auxin for callus induction in cereals [11]. In our experiments, the low and high 2,4-D concentrations in M1, M2, and M3 (1.5, 1, and 4 mg/l, respectively) made embryogenic callus induction challenging and decreased the regeneration capacity, while the moderate concentration of 2,4-D in M4 medium (2.5 mg/l) with 0.5 mg/l Kinetin was effective and helpful for the development of embryogenic calli and increasing the regeneration frequency. Moreover, the development of embryogenic callus has been further enhanced through the addition of glycine and L-asparagine to M4 medium. Previous studies [24] indicated that immature inflorescences are highly competent and may give a very high embryogenic response over a wide range of 2,4-D supplementation in the presence of kinetin. Likewise, the inclusion of amino acids in callus induction media has been shown to be a critical factor for embryogenic callus formation [18].

For regeneration, a well-balanced combination of auxins and cytokinins has been recommended in previous studies [22, 42] to enhance shoot and root induction and plant regeneration. In the present study, supplementing MS medium with a combination of kinetin (1 mg/l), Zeatin (0.5 mg/l), TDZ (0.1 mg/l), and ABA (0.25 mg/l) for shoot induction and the combination of IAA (1 mg/L) and kinetin (1 mg/l) for rooting allowed shoot and root induction and plant regeneration from Giza-1 and Giza-2.

The release of toxic phenolic compounds from plant tissues into the culture medium is one of the major factors affecting the in vitro culture of sorghum inflorescence, which causes browning in tissue culture and results in low levels of genetic transformation and regeneration frequencies [11, 12, 22]. In the present study, we observed that the tissue culture media supplemented with (0.3 g/L) PVP, charcoal (0.4 g/L), and ascorbic acid (10 mg/L) were able to reduce the quantity of phenolic compounds released into the medium during the subsequent subcultures of the regenerated cultures, and consequently, the medium’s browning was significantly reduced. This is in agreement with the previous observations in sorghum [12]. Furthermore, frequent transfer of the cultures onto fresh medium may inhibit a significant degree of phenolic compound accumulation in the medium, which is beneficial in the prevention of browning in plant tissue culture [12, 22].

Biolistic particle delivery system is a useful tool for delivering external DNA into plant tissues. In this respect, different authors optimized the physical and biological parameters to achieve higher levels of transformation efficiency [10, 15, 43]. However, based on our experience, we were able to recover transformed calli and regenerated plants using pre- and post-osmotic treatment of bombarded calli, 1100 psi acceleration pressure, and a 9-cm microcarrier flying distance [44].

Using the PCR technique as an indicator for the presence of transgenes in the genomic DNA of putatively transgenic plants has been reported by many investigators [27, 35, 45]. In the present study, PCR analysis was used to confirm the positive events that acquired the targeted transgenes. The presence of Cas9, NPTII, and the 35S promoter in T0-generation plants confirmed the successful transfer of the gRNA-Cas9 construct into the transgenic plants

We used CRISPR/Cas9 technology to demonstrate a downregulation of COMT1 in sudangrass as an efficient lignocellulosic biofuel and feedstock crop with a 10% editing efficiency. COMT1 is a critical gene that regulates lignin production in plants. The overall characteristics of the CRISPR/Cas9-mediated COMT1 mutant were comparable to a recent study assessing sorghum RNAi lines with the downregulation of COMT [31]. Similar results have been reported in other crops with reduced COMT activity, including sugarcane [35, 37], barely [31, 46], maize [47], rice [48], and switchgrass [49].

The development of genetically edited crops similar to those developed by conventional or mutation breeding using this potential technique makes it a promising tool for providing sustainable productive agriculture for better food for a rapidly growing population and animal feed, as well as biofuel in a changing climate condition [50, 51]. The approach presented here could be further developed to optimize the construction of Cas9/gRNA cassettes for genome editing in a variety of other plants. Furthermore, targeted COMT mutations will most likely increase the use of sudangrass as an important source of forage and biofuel production.

## Conclusion

The present study demonstrated the efficient development of a tissue-culture-based regeneration system in sudangrass using immature inflorescence explants from two cultivars, Giza-1 and Giza-2. The biolistic transformation system has been applied for efficient gene editing and targeted gene modification using the CRISPR/Cas9 system to demonstrate a downregulation of caffeic acid *O*-methyl transferase (COMT) as a key enzyme in the lignin biosynthetic pathway in an attempt to reduce lignin content and enhance forage and biomass quality in sudangrass germplasms. The developed plant regeneration system provides a foundation for the genetic transformation of sudangrass, which is of significant importance for improving economically important traits. This is the first research to report CRISPR/Cas9 gene editing in sudangrass. Further optimization of the protocol used in this study could be used for large-scale production of transgenic sudangrass. Further parameters could be applied to study the mutant inheritance in subsequent generations.

## Data Availability

The datasets generated during and/or analyzed during the current study are available from the corresponding author on reasonable request.

## Abbreviations

CRISPR: Clustered Regularly Interspaced Short Palindromic Repeats
Cas9: CRISPR-associated protein 9
MS: Murashige and Skoog medium
COMT: Caffeic acid O-methyl transferase
SDGs: Sustainable development goals
MES: 2-(*N*-morpholino)ethanesulfonic acid
2,4-D: 2,4-Dichlorophenoxyacetic acid
IAA: Indole-3-acetic acid
TDZ: Thidiazuron
BAP: Benzylaminopurine
ABA: Abscisic acid
PVP: Polyvinyl pyrrolidone
gRNA: Guide RNA
NPTII: Neomycin phosphotransferase II
PDS: Particle delivery system
PCR: Polymerase chain reaction
WTC: Wild type control
PAC: Positive amplification control
NTC: Negative template control
MSA: Multiple sequence alignment
PAM: Protospacer adjacent motif
